# Deep learning‐based auto‐contouring for organs at risk in three‐dimensional image‐guided brachytherapy for cervical cancer and endometrial cancer

**DOI:** 10.1002/acm2.70570

**Published:** 2026-04-09

**Authors:** Kirika Takahashi, Ken Takeda, Hisamichi Takagi, Akari Niiyama, Noriyuki Kadoya, Yoshiyuki Katsuta, Kazuhiro Arai, Shohei Tanaka, Noriyoshi Takahashi, Takaya Yamamoto, Rei Umezawa, Keiichi Jingu

**Affiliations:** ^1^ Department of Therapeutic Radiology Health Sciences Tohoku University Graduate School of Medicine Sendai Japan; ^2^ Department of Radiation Oncology Tohoku University Graduate School of Medicine Sendai Japan

**Keywords:** automatic segmentation, brachytherapy, cervical cancer, deep learning, endometrial cancer

## Abstract

**Background:**

Automatic contouring can reduce the time required for delineating organs at risk (OARs) in brachytherapy planning and minimize interobserver variability.

**Purpose:**

This study aimed to develop and evaluate a deep learning‐based automatic contouring model for OARs in three‐dimensional image‐guided brachytherapy (3D‐IGBT) for cervical and endometrial cancer, including cases with interstitial needles.

**Methods:**

The dataset comprised 100 patients (140 cases) with cervical or endometrial cancer who underwent 3D‐IGBT. Interstitial needles were used in 74 cases. The nnU‐Net model was trained (80 patients, 80 cases) and tested (20 patients, 60 cases). The OARs considered were the bladder, small bowel, rectum, and sigmoid. Ground truth (GT) contours were manually delineated by radiation oncologists and medical physicists. Evaluation included measuring inference time and assessing geometric agreement using dice similarity coefficient (DSC), surface DSC (sDSC), Hausdorff distance (HD), and 95th percentile HD (95HD). These metrics were also calculated for a combined structure of the rectum and sigmoid (Rec+Sig). Furthermore, D_2cc_ was calculated based on both the GT and predicted contours using the clinical dose distribution, and the difference between them (ΔD_2cc_) was evaluated. Differences in accuracy with or without interstitial needles were compared using Welch's *t*‐test (significance level: *p* < 0.05).

**Results:**

Mean processing time was 30.3 s per case. Mean DSC values for the bladder, small bowel, rectum, sigmoid, and Rec+Sig were 0.96, 0.79, 0.83, 0.76, and 0.87, respectively. Mean 95HD values (mm) were 4.01, 18.8, 13.6, 25.5, and 17.8, respectively; ΔD_2cc_ values (Gy) were 0.17, 0.53, 0.014, −0.073, and −0.045, respectively. No significant accuracy differences related to interstitial needles were observed for any of the OARs.

**Conclusions:**

The proposed deep learning model demonstrated potential for application in cases involving interstitial needles and may contribute to improving the efficiency of the treatment planning workflow.

## 1 INTRODUCTION

The incidence and mortality rates of both cervical and endometrial cancers have shown a growing trend in Japan in recent years.^1^, ^2^ In definitive radiotherapy for these cancers, brachytherapy plays an extremely important role, and recently, three‐dimensional image‐guided brachytherapy (3D‐IGBT) using CT or MRI has been recommended as a standard treatment.^3^ With its widespread adoption, patient‐specific dose prescriptions based on 3D images have become achievable. This has been shown to improve tumor control rates and reduce the incidence of late adverse events.^4^, ^5^ Further, in recent years, in addition to conventional intracavitary brachytherapy, combined intracavitary and interstitial brachytherapy (Hybrid BT), which combines interstitial irradiation, has been widely applied for cases involving massive tumors or asymmetrical infiltration.^6^

3D‐IGBT enables precise delivery of high‐dose radiation localized to the tumor. However, it requires strict management of dose constraints to organs at risk (OARs). In brachytherapy, the proximity of radiation sources to organs results in steep dose gradients near contour boundaries; thus, even slight inaccuracies in organ delineation can significantly affect dose uncertainty.^7^ Kirisits et al. reported that uncertainty in the maximum dose and D_2cc_ can amount to approximately 9%.^8^ Therefore, accurate contouring of both the OARs and the clinical target volume is essential for appropriate dose assessment. In addition, because treatment planning in 3D‐IGBT is performed while the applicator remains inserted in the patient, positional changes of internal organs or the applicator itself could occur if planning is prolonged.^9^, ^10^, ^11^ Thus, it is necessary to accelerate treatment planning.

However, the treatment planning workflow in 3D‐IGBT involves time‐consuming manual processes, particularly for OAR delineation. As mentioned above, OAR delineation is a critical procedure in brachytherapy; however, simultaneously achieving speed and accuracy in this process through manual contouring is challenging. Moreover, it is known to vary depending on the experience and knowledge of the operator performing the contouring. Saarnak et al. reported an interobserver variability of 10%–11% in bladder and rectum D_2cc_ values based on CT images.^7^ Furthermore, unlike conventional external beam radiotherapy (EBRT), it is necessary to perform real‐time treatment planning for 3D‐IGBT. Consequently, it requires the simultaneous involvement of multiple specialized staff members, which is also challenging. A time‐driven activity‐based costing analysis comparing brachytherapy to EBRT for cervical cancer demonstrated that the former required higher costs and significantly more time from both radiation oncologists and medical physicists,^12^, ^13^, ^14^ highlighting the substantial workload placed on clinical staff.

As a potential solution to these challenges, the application of deep learning‐based automatic contouring technologies to the treatment planning process is being explored. In recent years, the rapid advancement of deep learning‐based automatic segmentation techniques for medical imaging has prompted efforts to perform automatic contouring in 3D‐IGBT cases.^15^, ^16^, ^17^, ^18^, ^19^ This approach has the potential to standardize and enhance the reproducibility of contouring, accelerate treatment planning, and reduce the burden on clinical staff. However, previous studies have focused mainly on cases using tandem‐ovoid applicators. Consequently, the applicability of this technology to cases using multiple applicator types—such as cylinders and tandem‐cylinders—as well as to Hybrid BT cases that incorporate interstitial needles for more complex dose distributions, has not yet been sufficiently investigated. Addressing the diversity of applicator types and Hybrid BT cases is clinically significant. This is because it contributes to improved robustness and efficiency of workflow across a broader spectrum of clinical settings.

In this study, we aimed to develop a deep learning‐based automatic contouring model for OARs in 3D‐IGBT cases of cervical and endometrial cancer including cases of Hybrid BT. For the deep learning model, we adopted nnU‐Net, a self‐configuring method for deep learning‐based image segmentation frameworks.^20^ Although the application of nnU‐Net to gynecological pelvic 3D‐IGBT treatment planning involving applicators and needles has been limited, the model has demonstrated superior performance compared to many existing segmentation methods in other domains, suggesting its strong potential to address the challenges.^20^ This study aimed to develop a robust automatic contouring model applicable even to Hybrid BT cases using interstitial needles and to improve its accuracy for clinical application.

## 2 METHODS

### 2.1 Clinical dataset

This study analyzed treatment planning CT images obtained from 100 patients (140 cases) with cervical or endometrial cancer who underwent multiple sessions of 3D‐IGBT at Tohoku University Hospital between July 2022 and February 2024. As shown in Table 1, 80 cases, one fraction per patient from 80 patients, were used as the training dataset, while 60 cases covering all treatment fractions (three fractions per patient) from 20 patients were used as the test dataset. All patients received EBRT to the whole pelvis at a dose of 45–50.4 Gy in 25–28 fractions, including those who received a boost irradiation of 9–12.6 Gy in 5–7 fractions. Following EBRT, 3D‐IGBT was administered at either 2 or 3 fractions. The dataset also included cases in which patients underwent 3D‐IGBT alone, with a total dose of six fractions. The applicators and their respective case counts (Table 1), including the number of cases with interstitial needles, were as follows: tandem‐ovoids (*N* = 104; including 59 with interstitial needles), cylinders (*N* = 20; 6), tandem‐cylinders (*N* = 13; 9), and metal applicators (*N* = 3; 0). The tandem‐ovoids were performed using a standard CT/MR Applicator, the cylinders using a vaginal CT/MR Applicator, the tandem‐cylinders using a modified intrauterine tube inside the cylinder, and the needles using ProGuide Needles, Obturators and Markers (Nucletron B.V. Veenendaal, Netherlands). Additionally, contrast agents were not routinely used for the bladder according to our institutional protocol; therefore, cases in which contrast agents were exceptionally administered were excluded from the analysis. All CT images were acquired using an Aquilion/LB scanner (Toshiba Medical Systems, Japan) and reconstructed with a matrix size of 512 × 512–pixel, an in‐plane resolution of 0.977 mm × 0.977 mm and a slice thickness of 2 mm.

**TABLE 1:** Composition of the clinical dataset.

	Applicator	Interstitial needle	Patients (*N*’ = 100)	Cases (*N* = 140)
Training data (*N* = 80)	Tandem & Ovoid	(−)	24	24
		(+)	32	32
	Cylinder	(−)	8	8
		(+)	6	6
	Tandem Cylinder	(−)	1	1
		(+)	6	6
	Metal	(−)	3	3
		(+)	–	–
Testing data (*N* = 60)	Tandem & Ovoid	(−)	7	21
		(+)	9	27
	Cylinder	(−)	2	6
		(+)	–	–
	Tandem Cylinder	(−)	1	3
		(+)	1	3
	Metal	(−)	–	–
		(+)	–	–

Interstitial needle (−) = cases without interstitial needles; (+) = cases with interstitial needles. Training data: 80 cases = 80 patients x 1 fraction; Testing data: 60 cases = 20 patients x 3 fraction.

The OARs targeted for contouring were the bladder, small bowel, rectum, and sigmoid. Clinically used contour data were subjected to quality control by one medical physicist and two radiological technologists. The contours subsequently approved by a radiation oncologist were defined as the ground truth (GT) in this study. The dose distributions clinically generated by medical physicists and radiation oncologists were used without modification. Contouring was performed using Eclipse version 13.60 or version 18.00 (Varian Medical Systems, Palo Alto, CA, USA), while dose calculation was carried out using Oncentra Brachy version 4.5.5.23 (Elekta, Stockholm, Sweden). At our institution, contouring is performed in Eclipse, after which the contour data (RT‐Structure) are transferred to Oncentra, where dose calculation and evaluation are conducted based on the transferred contours.

This study was approved by the Ethics Committee Tohoku University Graduate School of Medicine (Approval No. 2025‐1‐222).

### 2.2 Workflow

**FIGURE 1:**
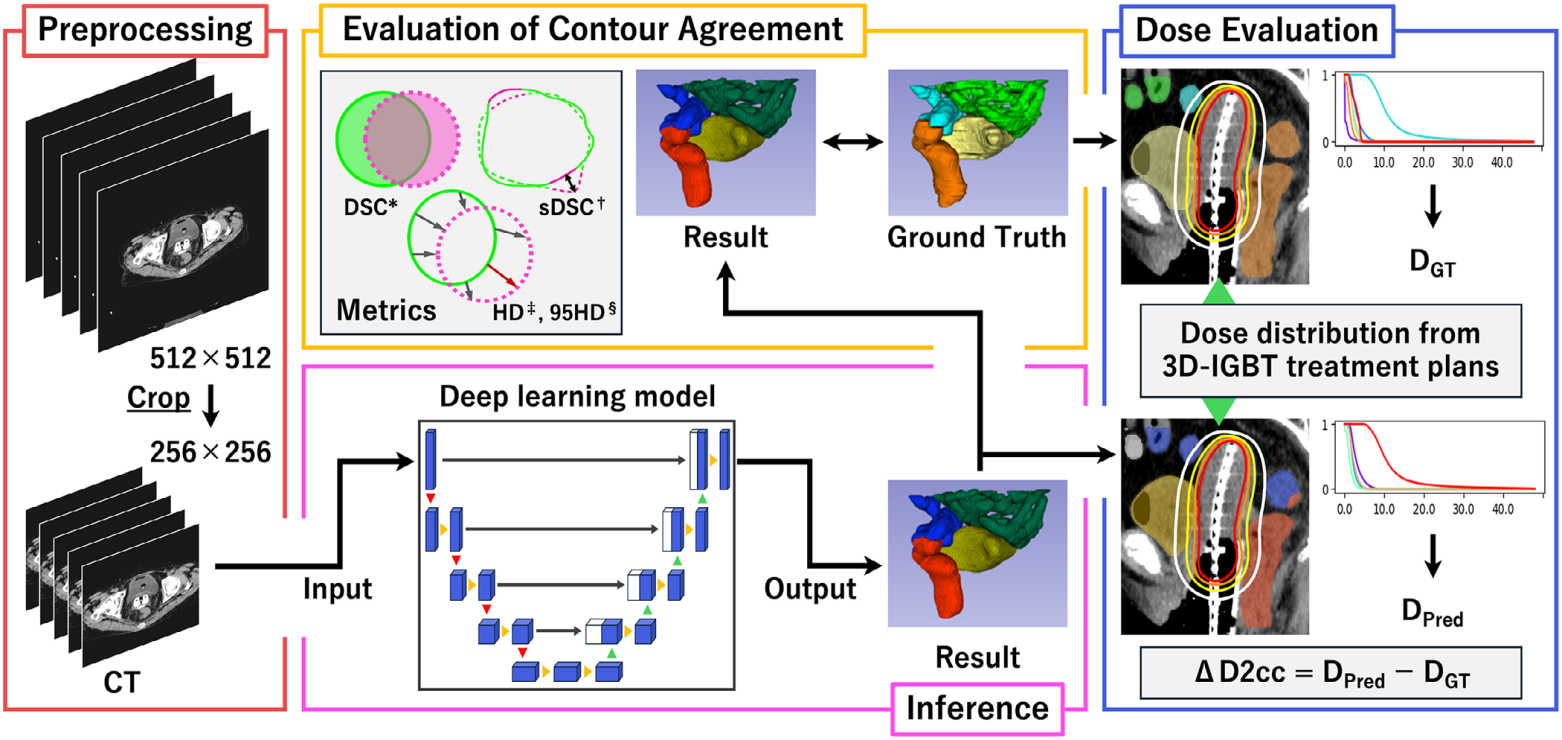
Workflow of this study. A deep learning‐based automatic contouring model was developed using treatment planning CT images as input and generating contours of OARs as output. To evaluate the agreement between the GT and predicted contours, we used the dice similarity coefficient (DSC^*^), surface dice similarity coefficient (sDSC^†^), Hausdorff distance (HD^‡^), and 95^th^‐percentile Hausdorff distance (95HD^§^). Furthermore, the dose distributions from 3D‐IGBT treatment plans were applied to the GT and predicted contours to evaluate the D_2cc_ for each OAR, both on a per‐fraction basis and as a three‐fraction cumulative total.

The workflow of this study is illustrated in Figure 1. The model architecture used in this study was based on the nnU‐Net framework.

### 2.3 Input data preprocessing

At a resolution of 512 × 512–pixel, the original CT images were large and computationally intensive. Therefore, the images were cropped to 256 × 256–pixel to ensure the inclusion of all target OARs. Image cropping was performed by extracting a 256 × 256–pixel region centered on the central coordinates of each CT image, with pixels outside this region removed. To avoid resolution loss due to downsampling, the pixel size was kept unchanged. Additionally, the number of slices per case was standardized to 128. For cases with more than 128 slices, the most caudal 128 slices were selected, and the remaining slices outside this range were excluded. For cases with fewer than 128 slices, blank slices (pixel value = 0) were added to achieve consistent input data sizes. The CT image density settings were as follows: a window width of 400, a window level of 0. In addition, 8‐bit images were used. The contour information of the OARs was extracted from Digital Imaging and Communications in Medicine (DICOM) radiotherapy (RT) Structure Set files to create training labels. All DICOM files containing the contour information were converted to Neuroimaging Informatics Technology Initiative format. Pydicom version 2.3.1 was used for DICOM file reading/writing, and rt‐utils version 1.2.7 was used for DICOM‐RT Structure Set format conversion. All processing was performed in an in‐house environment using Python 3.8.10.

### 2.4 Deep learning model

For the deep learning model, we used nnU‐Net version 1.2.0, which is a self‐configuring method for biomedical image segmentation developed by Isensee et al.^20^ In this study, on the basis of the report by Duprez et al.,^18^ the 3D full‐resolution U‐Net, which demonstrated the highest accuracy among the three U‐Net architectures provided by nnU‐Net, was selected for model training. nnU‐Net performed 5‐fold cross‐validation for model training, and the training parameters were automatically optimized. The five trained models were ensembled using the default averaging method to create a single trained model.

In addition, OAR contouring was inferred using the trained model, and the inference time was measured. Inference time was measured by processing each case sequentially and recording the total processing time, as well as the time for each individual step, including input data preprocessing (DICOM files loading, image cropping, intensity normalization, and data type conversion), inference (contouring), and postprocessing (exporting and saving DICOM files). Data transfer time was excluded from the analysis. Timing was recorded using recording execution timestamps using the Python datetime module, and durations were calculated as the differences between the start and end times of each processing step. The measurement was conducted in a computing environment equipped with an Intel Core i7‐14700KF CPU and an Nvidia GeForce RTX 4090 GPU (24 GB).

### 2.5 Evaluation of contouring accuracy

The contouring accuracy of the nnU‐Net model was evaluated using the 60 cases in the test set (Table 1). For each OAR, the inferred contours were compared with the GT contours. To eliminate the influence of interobserver variability in the contouring of the rectum and sigmoid and to enable a more objective evaluation, a new structure was created in both the GT and predicted contours by merging the rectum and sigmoid into a single structure (hereinafter referred to as “Rec+Sig”). An accuracy evaluation was also performed for this combined structure.

The evaluation metrics included the DSC,^21^ the sDSC,^22^ the HD, and the 95HD.^23^ The DSC is one of the most commonly used metrics for evaluating segmentation accuracy, assessing the overall volumetric overlap between the GT and predicted contours. The sDSC evaluates the overlap of the contour surfaces (boundaries) between the GT and predicted contours within a specified tolerance (in mm). In this study, a tolerance of 2 mm was adopted, referencing the value applied in a previous study on automatic abdominal contouring by Amjad et al.^24^ The HD assesses the maximum distance between the boundaries of the GT and predicted contours. Since the HD is sensitive to outliers, we also employed the more robust 95HD, which represents the 95th percentile of the distances.

### 2.6 Dose evaluation

Dose evaluation was performed for all 60 test cases to assess the impact of the inferred OAR contours on dosimetric parameters. Using the clinically planned DICOM‐RT Dose files, the D_2cc_ values were calculated for both the GT and predicted contours on the same coordinate system.

These processes were performed using PlatiPy version 0.7.2 (https://pypi.org/project/platipy/), a Python‐based medical image analysis library. Clinical dose constraints were defined using the summed dose of EBRT and 3D‐IGBT, converted to EQD2 (*α*/*β* = 3), as follows: bladder, <90 Gy; small bowel, <70 Gy; rectum and sigmoid, <75 Gy.

Dose evaluation was performed on a per‐fraction basis for all 60 cases and for cumulative dose assessment across all 20 patients (EBRT: 45 Gy in25 fractions + 3D‐IGBT: 18 Gy in three fractions). For the per‐fraction evaluation, D_2cc_ derived from the GT contours (D_GT_) and from the predicted contours (D_Pred_) were compared, and the ΔD_2cc_ was calculated using the following equation:

$$\;\Delta D_{2cc}=D_{Pred}\;-D_{GT}$$

For cumulative dose evaluation, the summed dose over three fractions for each patient of D_2cc_ (D_GT_ and D_Pred_) was converted to EQD2 (EQD2_GT_ and EQD2_Pred_) using the following equation, and the resulting values were then compared with the predefined dose constraints.

$$EQD2=\sum_{k=1}^{3}\frac{d_{k}\cdot 1\cdot \left(d_{k}+\alpha /\beta \right)}{\left(2+\alpha /\beta \right)}$$

(d_k_: D_2cc_ for fractions 1–3; α/β = 3)

Furthermore, using the converted EQD2_GT_ and EQD2_Pred_ values along with the dose constraints, cumulative dose differences were evaluated by calculating ΔEQD2 (%) according to the following equation:

$$\Delta EQD2\;\left(\%\right)=\frac{EQD2_{Pred}-EQD2_{GT}}{Dose\;constraint}$$

### 2.7 Statistical analysis

To evaluate the impact of interstitial needles on contouring accuracy, the 60 test cases (20 patients) were divided into the needle group (10 patients, 30 cases) and the non‐needle group (10 patients, 30 cases). The two groups were compared in terms of geometric evaluation metrics (DSC, sDSC, HD, and 95HD) and ΔD_2cc_ using the Welch's *t*‐test. In addition, to assess the overall dosimetric accuracy based on the predicted contours, the paired *t*‐test was used to compare D_GT_ and D_Pred_ for all 60 test cases. For all statistical analyses, a *p* value of <0.05 indicated statistically significant differences.

## 3 RESULTS

### 3.1 Time required for contouring

For the 60 test cases, processing time was measured on a per‐case basis. The mean total processing time per case was 30.3 s, including input data preprocessing (DICOM files loading, image cropping, intensity normalization, and data type conversion), inference (contouring), and postprocessing (exporting and saving DICOM files). The mean processing times per case for preprocessing, inference, and postprocessing were 1.26, 27.9, and 1.17 s, respectively.

### 3.2 Quantitative evaluation of contour agreement: overall results

Table 2 summarizes the mean and standard deviation of the evaluation metrics (DSC, sDSC, HD, 95HD) for each OAR (bladder, small bowel, rectum, sigmoid, Rec+Sig) across all 60 test cases. The contouring accuracy of the nnU‐Net model showed the highest agreement with the GT for the bladder, with minimal inter‐case variability. The mean DSC exceeded 0.8 for the bladder (0.96 ± 0.018), rectum (0.83 ± 0.071), and Rec+Sig (0.87 ± 0.067). Similarly, the mean sDSC exceeded 0.8 for all OARs except the sigmoid (bladder: 0.97 ± 0.031, small bowel: 0.83 ± 0.16, rectum: 0.82 ± 0.094, Rec+Sig: 0.88 ± 0.091). Conversely, regarding HD and 95HD, substantial inter‐case variability was observed, with mean values exceeding 10 and 5 mm, respectively, for all OARs except the bladder.

**TABLE 2:** Quantitative evaluation of automated contouring and comparison with previous studies.

		Organ				
Study	Parameter	Bladder	Small bowel	Rectum	Sigmoid	Rec+Sig
Current study	DSC	0.96 ± 0.018	0.79 ± 0.17	0.83 ± 0.071	0.76 ± 0.14	0.87 ± 0.067
	sDSC	0.97 ± 0.031	0.83 ± 0.16	0.82 ± 0.094	0.78 ± 0.15	0.88 ± 0.091
	HD (mm)	9.69 ± 11.8	33.5 ± 19.9	20.5 ± 11.3	36.5 ± 21.8	32.7 ± 24.3
	95HD (mm)	4.01 ± 8.00	18.8 ± 17.0	13.6 ± 9.77	25.5 ± 20.9	17.8 ± 20.7
Zhang et al.^15^	DSC	0.87 ± 0.032	0.80 ± 0.058	0.82 ± 0.050	0.65 ± 0.079	
	HD (mm)	12.1 ± 4.0	27.8 ± 10.8	19.6 ± 8.7	19.6 ± 8.7	
Mohammadi et al.^16^	DSC	0.96 ± 0.037		0.97 ± 0.015	0.93 ± 0.033	
	95HD (mm)	2.30 ± 3.37		1.42 ± 1.41	2.10 ± 1.36	
Jiang et al.^17^	DSC	0.86 ± 0.086	0.56 ± 0.13	0.86 ± 0.089	0.66 ± 0.12	
	HD (mm)	19.8 ± 11.4	68.1 ± 33.8	12.3 ± 8.08	98.4 ± 51.0	
Duprez et al.^18^	DSC	0.92 ± 0.04		0.84 ± 0.04		
	95HD (mm)	3.00 ± 1.09		5.25 ± 1.78		
Lei et al.^19^	DSC	0.83 ± 0.10	0.63 ± 0.22	0.76 ± 0.15	0.64 ± 0.16	
	95HD (mm)	7.82 ± 4.85	23.0 ± 20.4	18.9 ± 22.6	23.0 ± 15.2	

Rec+Sig is a structure uniquely defined in this study that was created by merging the rectum and sigmoid. DSC, dice similarity coefficient; sDSC, surface dice similarity coefficient; HD, Hausdorff distance; 95HD, 95th‐percentile Hausdorff distance.

**FIGURE 2:**
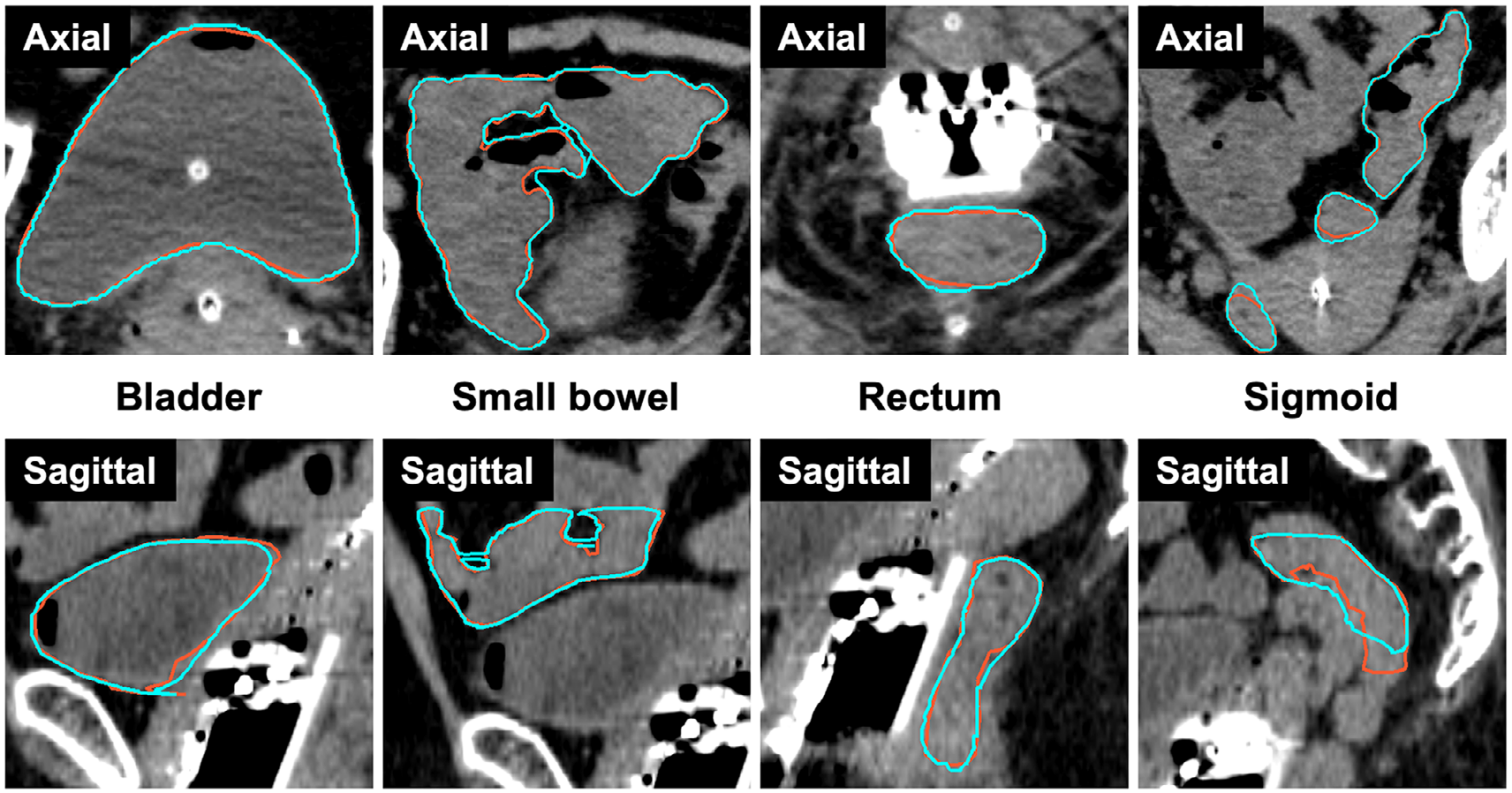
Example with relatively high agreement between the GT and the predicted contours. The contour lines are shown in orange for the GT and in cyan for the predicted contours. Overall, the predicted contours generally showed high agreement with the GT contours.

**FIGURE 3:**
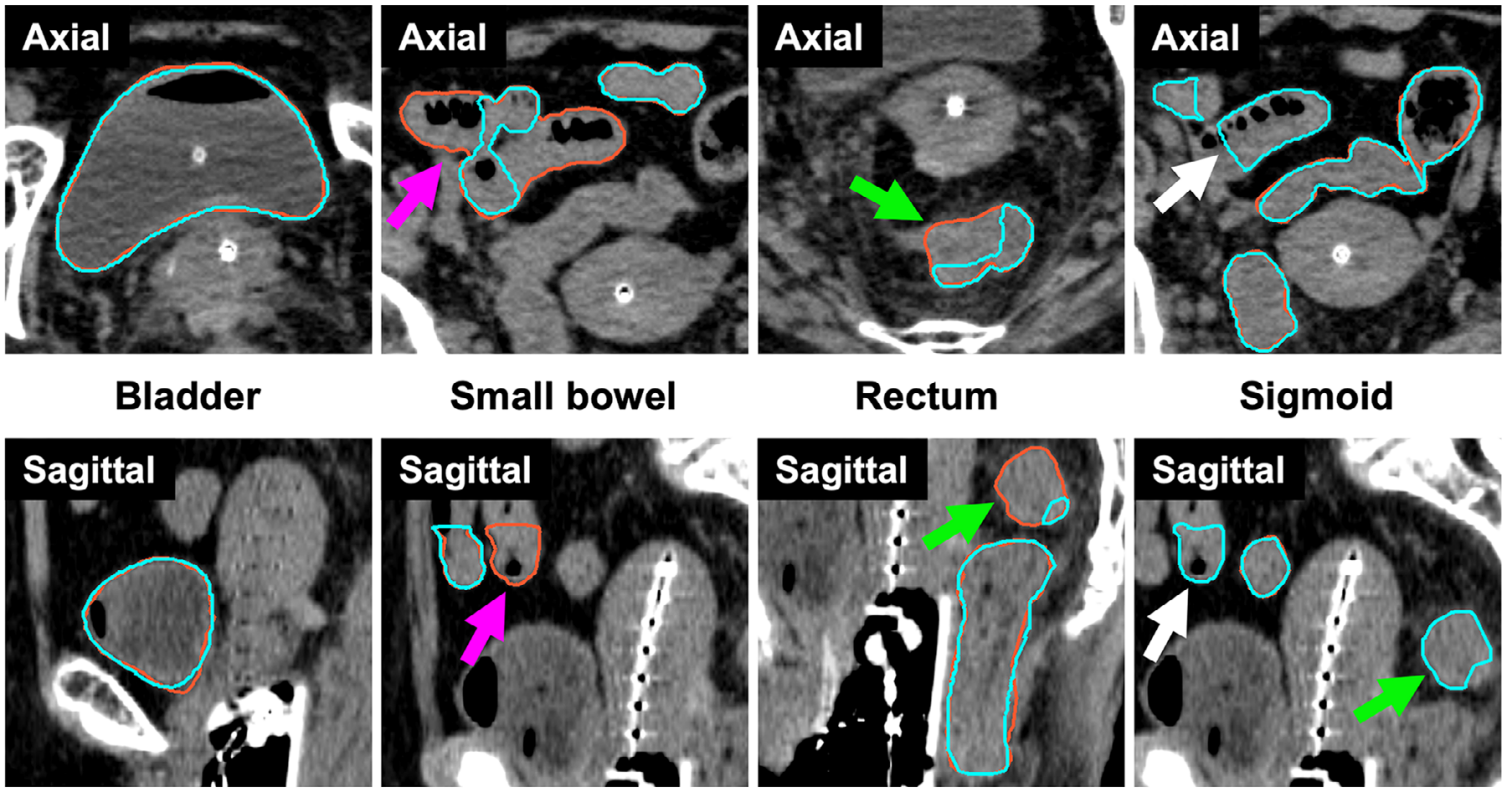
Example with relatively low agreement between the GT and the predicted contours. The contour lines are shown in orange for the GT and in cyan for the predicted contours. As indicated by the magenta arrows and the white arrows, the small bowel and sigmoid were not correctly segmented (magenta: failure to contour the small bowel; white: misclassification of the small bowel as the sigmoid). Additionally, as shown by the green arrows, misclassification was observed at the rectosigmoid junction.

Figure 2 shows cases in which the agreement between the GT and predicted contours was relatively high, while Figure 3 shows those in which this agreement was relatively low. In Figure 2, the evaluation metrics for this case were as follows: DSC—bladder: 0.97, small bowel: 0.95, rectum: 0.90, sigmoid: 0.92; 95HD (mm)—bladder: 1.99, small bowel: 4.0, rectum: 1.26, sigmoid: 2.19. Although some slices in the rectosigmoid junction were not properly segmented, the overall contours were broadly consistent. In contrast, Figure 3 shows that segmentation errors occurred not only at the rectosigmoid junction but also in the small bowel and sigmoid, spanning a wide range of slices. The evaluation metrics for this case were as follows: DSC—bladder: 0.96, small bowel: 0.66, rectum: 0.84, sigmoid: 0.74; 95HD (mm)—bladder: 2.19, small bowel: 20.8, rectum: 20.6, sigmoid: 45.7.

### 3.3 Quantitative evaluation of contour agreement: needle group vs. non‐needle group

**FIGURE 4:**
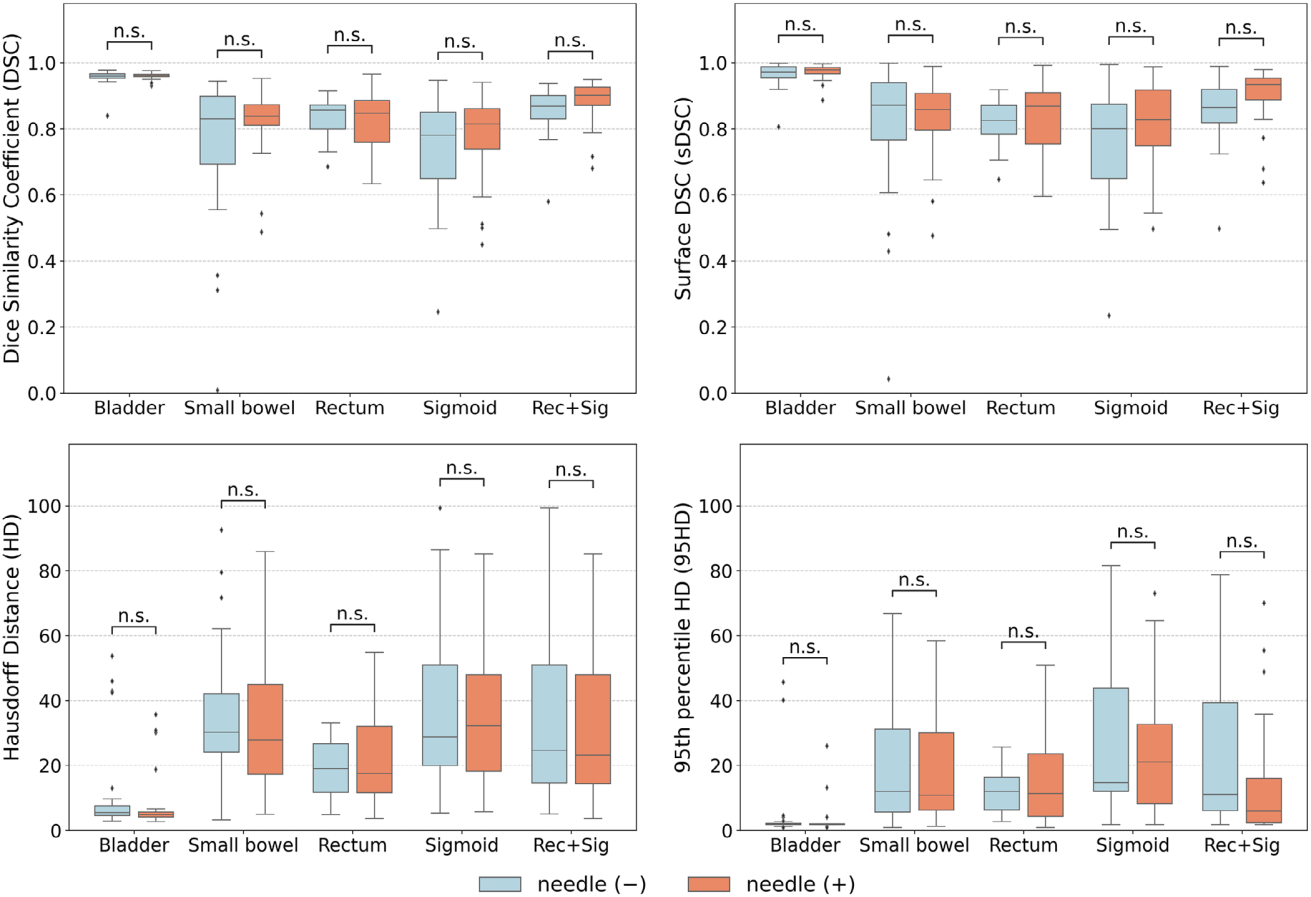
Box plots comparing accuracy based on the presence or absence of interstitial needles. Box plots corresponding to the results in Table 3. “n.s.” indicates no significant difference (*p* > 0.05); for items where a significant difference was observed, the calculated p value is displayed.

Table 3 and Figure 4 present the investigation of accuracy differences based on the presence or absence of combined interstitial needles. A Welch's *t*‐test performed between the needle group and the non‐needle group. No statistically significant differences were observed for any evaluation metric across all OARs (*p* > 0.05). However, for Rec+Sig, the *p*‐values for DSC, sDSC, and 95HD were relatively smaller, indicating a trend toward improved performance in the needle group.

**TABLE 3:** Results of an accuracy comparison based on the presence or absence of interstitial needles.

		Organ				
Parameter		Bladder	Small bowel	Rectum	Sigmoid	Rec+Sig
DSC	Needle (−)	0.96 ± 0.023	0.76 ± 0.21	0.83 ± 0.056	0.74 ± 0.15	0.85 ± 0.071
	Needle (+)	0.96 ± 0.010	0.82 ± 0.10	0.82 ± 0.084	0.78 ± 0.13	0.89 ± 0.060
	*p‐*value	0.36	0.17	0.66	0.23	0.071
sDSC	Needle (−)	0.96 ± 0.038	0.81 ± 0.20	0.82 ± 0.065	0.76 ± 0.16	0.86 ± 0.095
	Needle (+)	0.97 ± 0.022	0.84 ± 0.12	0.83 ± 0.12	0.81 ± 0.13	0.91 ± 0.082
	*p*‐value	0.26	0.57	0.74	0.13	0.067
HD (mm)	Needle (−)	11.1 ± 14.0	34.3 ± 20.9	19.5 ± 8.07	38.1 ± 22.8	34.5 ± 25.5
	Needle (+)	8.28 ± 8.78	32.7 ± 18.8	21.5 ± 13.7	35.0 ± 20.7	31.0 ± 22.9
	*p*‐value	0.36	0.76	0.50	0.58	0.59
95HD (mm)	Needle (−)	4.87 ± 10.2	19.9 ± 18.9	12.2 ± 6.45	27.6 ± 21.9	22.5 ± 22.6
	Needle (+)	3.16 ± 4.72	17.7 ± 14.7	14.9 ± 12.1	23.4 ± 19.6	13.2 ± 17.3
	*p‐*value	0.42	0.62	0.29	0.45	0.085

Rec+Sig is a structure uniquely defined in this study that was created by merging the rectum and sigmoid. DSC, dice similarity coefficient; sDSC, surface dice similarity coefficient; HD, Hausdorff distance; 95HD, 95th‐percentile Hausdorff distance. Needle (−) = the non‐needle group; (+) = the needle group. There were no statistically significant differences between the two groups in any of the OARs. For Rec+Sig, the needle (+) group showed trends toward improvements in the DSC, sDSC, and 95HD.

### 3.4 Dose evaluation: overall results

Table 4 summarizes the per‐fraction dose evaluation results for all 60 test cases, including D_2cc_ of the D_2cc_ of the GT contours (D_GT_, Gy), the D_2cc_ of the predicted contours (D_Pred_, Gy), and their difference (ΔD_2cc_, Gy). The paired *t*‐test revealed no statistically significant differences between D_GT_ and D_Pred_ for all OARs except for the small bowel (*p* > 0.05), indicating that D_Pred_ was generally comparable to D_GT_. For the small bowel, however, D_Pred_ was significantly higher than D_GT_ (*p* < 0.05).

**TABLE 4:** Results of the dosimetric evaluation per fraction.

		Organ				
Parameter		Bladder	Small bowel	Rectum	Sigmoid	Rec+Sig
D_GT_		5.98 ± 0.71	3.05 ± 1.22	5.38 ± 0.58	4.44 ± 1.02	5.16 ± 0.45
D_Pred_		6.15 ± 0.70	3.53 ± 1.16	5.40 ± 0.60	4.36 ± 0.93	5.12 ± 0.49
*p*‐value		0.19	0.031	0.90	0.68	0.61
ΔD_2cc_	All	0.17 ± 0.37	0.53 ± 0.90	0.014 ± 0.42	−0.073 ± 0.51	−0.045 ± 0.32
	Needle (−)	0.13 ± 0.37	0.56 ± 1.02	0.02 ± 0.46	−0.11 ± 0.51	0.0053 ± 0.37
	Needle (+)	0.22 ± 0.35	0.51 ± 0.77	0.0058 ± 0.37	−0.039 ± 0.51	−0.095 ± 0.26
	*p*‐value	0.32	0.84	0.88	0.62	0.24

Rec+Sig is a structure uniquely defined in this study that was created by merging the rectum and sigmoid. D_GT_, D_2cc_ based on the ground truth contours; D_Pred_, D_2cc_ based on the predicted contours. There were no statistically significant differences between D_GT_ and D_Pred_ in any of the OARs except the small bowel. ΔD_2cc_ = D_Pred_ − D_GT_. There were no statistically significant differences in any of the OARs between the non‐needle (−) and needle (+) groups.

**FIGURE 5:**
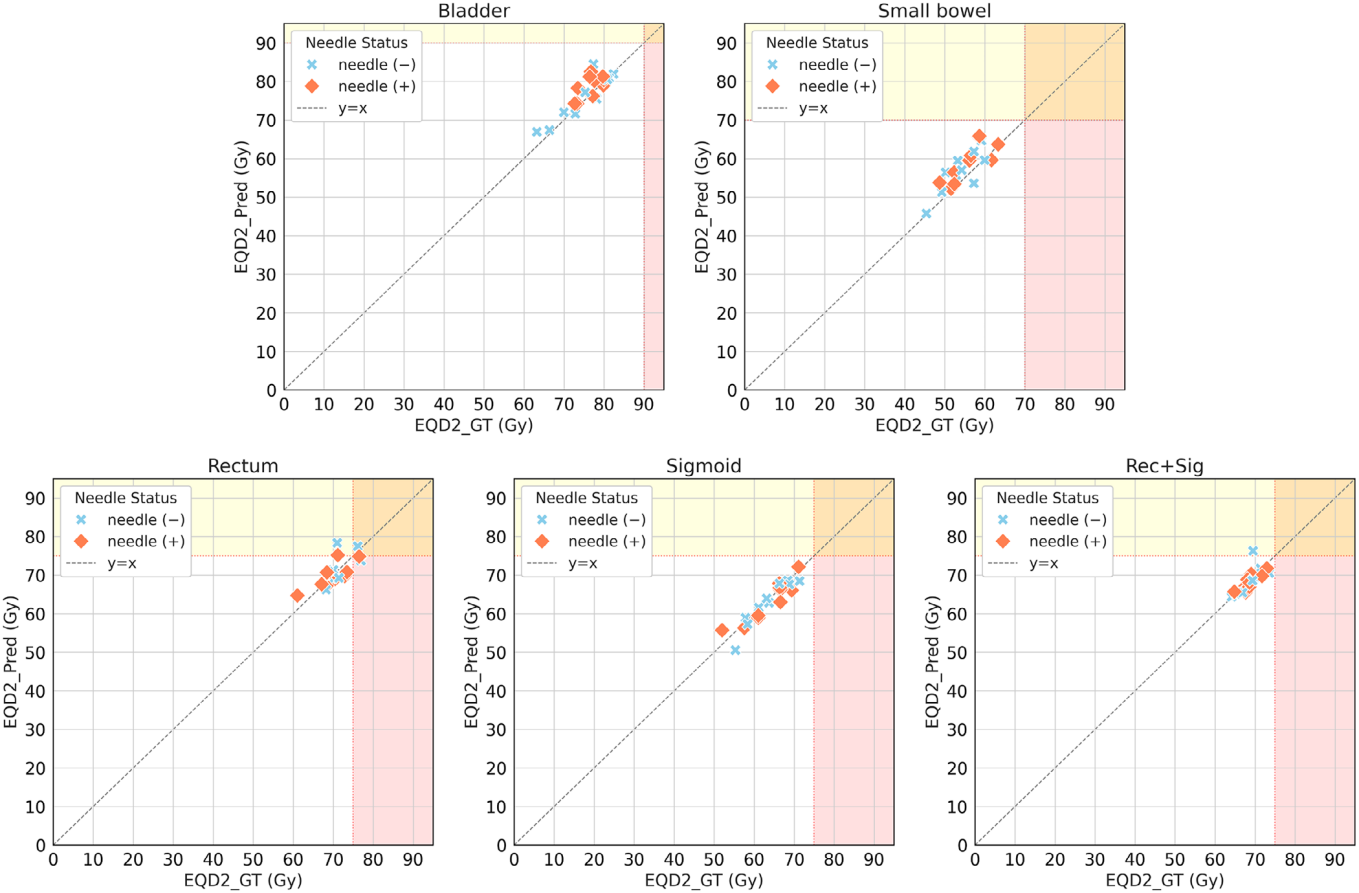
Scatter plot of cumulative EQD2 values based on ground truth and predicted contours. The *x*‐axis represents EQD2 based on GT (EQD2_GT_), and the *y*‐axis represents EQD2 based on predicted contours (EQD2_Pred_). The closer the points are to the *y* = *x* line (gray dotted line), the smaller the ΔEQD2 ( = EQD2_Pred_—EQD2_GT_). The white background area: both EQD2_GT_ and EQD2_Pred_ < constraints, the red area: EQD2_GT_ > constraints, the yellow area: EQD2_Pred_ > constraints, and the orange area: both >constraints. There were no statistically significant differences between the non‐needle (−) and needle (+) groups for all OARs.

The relationship between EQD2_GT_ and EQD2_Pred_ for cumulative dose evaluation is illustrated in the scatter plot (Figure 5). Overall, data points were densely clustered around the *y* = *x* line (EQD2_GT_ = EQD2_Pred_). For the bladder, small bowel, and sigmoid, all cases satisfied the dose constraints for both EQD2_GT_ and EQD2_Pred_. In contrast, some cases exceeded the dose constraints for the rectum and Rec+Sig. The dose differences ΔEQD2 (%) were as follows: bladder, 1.73 ± 2.81%; small bowel, 4.10 ± 4.26%; rectum, 0.17 ± 3.57%; sigmoid colon, −0.83 ± 2.69%; and Rec+Sig, −0.44 ± 2.63%. Although ΔEQD2 tended to be slightly higher for the bladder and small bowel compared to other OARs, no cases exceeded the prescribed dose constraints.

### 3.5 Dose evaluation: needle versus non‐needle group

Table 4 presents the comparison of ΔD_2cc_ (Gy) based on the presence or absence of interstitial needles. According to the Welch's *t*‐test, the needle group and the non‐needle group did not significantly differ in terms of ΔD_2cc_ due to needle usage in any OAR (*p* > 0.05). Even for Rec+Sig, which showed a trend toward improved geometric performance (DSC, sDSC, and 95HD) in the needle group, no significant difference was observed in ΔD_2cc_ (Gy). Similarly, no statistically significant differences in ΔEQD2 for cumulative dose evaluation were observed between cases with and without interstitial needle insertion for all OARs (*p* > 0.05).

## 4 DISCUSSION

Contouring of OARs is an essential step in providing appropriate dose delivery and minimizing radiation dose in 3D‐IGBT. However, manual contouring is time‐consuming and subject to inter‐ and intra‐observer variability. Considering that 3D‐IGBT requires real‐time treatment planning, there is significant demand for rapid and accurate contouring within the limited interval between applicator positioning and treatment initiation. In this study, an automated contouring model was developed using nnU‐Net for 3D‐IGBT cases, including those with combined interstitial needles (Hybrid BT), and its utility was verified. The mean processing time, including automated contouring, was 30.3 s per case, significantly reducing the time required compared with manual operation. This finding indicates the model's potential to contribute to workflow efficiency in real‐time treatment planning.

In the geometric evaluation of contours (Table 2), the model exhibited the highest accuracy for the bladder (DSC: 0.96, sDSC: 0.97, HD: 9.7 mm, and 95HD: 4.0 mm). This is likely attributed to the bladder's relatively high visibility on CT scan images and its regular shape. Although a relatively high accuracy was also achieved for the rectum (DSC: 0.83, sDSC: 0.82, HD: 20.5 mm, and 95HD: 13.6 mm), it was inferior to that of the bladder. The factors contributing to this may include difficulties in identifying the rectosigmoid junction and the inherent interobserver variability contained within the GT data itself. Conversely, the small bowel and sigmoid, which are highly mobile and complex in shape, were more likely to show a lower accuracy than other OARs (small bowel/sigmoid—DSC: 0.79/0.76, 95HD: 18.8/25.5 mm). For the sigmoid, difficulties in identifying boundaries not only at the junction with the rectum but also with the small bowel in contact with the ventral side affected the results. In the evaluation using Rec+Sig, eliminating uncertainty at the junction resulted in improved DSC and sDSC compared with single‐organ evaluations (DSC: 0.87, sDSC: 0.88). However, no significant improvement was observed in HD and 95HD (HD: 32.7 mm, 95HD: 17.8 mm). This is likely because, even after combination, local mismatches in the sigmoid continued to influence the maximum distance metrics.

Our results were compared with those of previous studies on CT scan‐based deep learning methods by Zhang et al.,^15^ Mohammadi et al.,^16^ Jiang et al.,^17^ Duprez et al.,^18^ and Lei et al.^19^ Table 2 shows the geometric accuracies reported in these studies. Notably, the studies differed in terms of datasets, deep learning models, applicator types, and the use of interstitial needles. In the geometric evaluation, the DSC of our model was generally comparable to that of previous studies.^15^, ^17^, ^18^, ^19^ Notably, the bladder accuracy was higher than that of other OARs, which is consistent with previous reports, which showed stable results. However, compared with the study of Zhang et al.,^15^ the accuracy (DSC, HD) for the small bowel in this study was slightly inferior. This discrepancy may be attributed to the differences in contouring method. In particular, Zhang et al. included not only the bowel lumen but also the inter‐bowel fat. Meanwhile, the current study focused solely on the bowel lumen. Further, compared with the research of Mohammadi et al.,^16^ our study showed a lower accuracy for the rectum and sigmoid. A contributing factor could be the absence of the small bowel in the participants. Based on our results, misclassifications were observed in regions where the sigmoid and small bowel were in contact. The simultaneous presence of the small bowel, which presents similar contrast, could have increased the difficulty of model training and inference. In addition, this study utilized cases with multiple types of applicators. Strict statistical comparison was not performed due to unbalanced sample sizes. However, there was no significant decrease in the accuracy of specific applicators. This finding indicates that the model possesses a certain degree of versatility across diverse applicators.

In the dosimetric evaluation per fraction (Table 4), the mean ΔD_2cc_ values were relatively low for the rectum (0.014 Gy) and Rec+Sig (−0.045 Gy). Considering that these OARs had a relatively high geometric accuracy, this finding shows a trend where geometric agreement contributes to reducing dosimetric errors. For the bladder, although geometric agreement of the contours was the highest among all OARs, ΔD_2cc_ was slightly larger. This may be explained by the fact that, in steep high‐dose gradient regions near the radiation source, even minimal boundary discrepancies can significantly impact D_2cc_. Consequently, minor contour deviations at the interface with the uterus or the high‐risk clinical target volume (HR‐CTV) may have resulted in relatively larger dose differences (ΔD_2cc_). Similarly, the small bowel, which exhibited comparatively lower geometric accuracy, tended to show larger ΔD_2cc_ values (0.53 Gy). This can be attributed to the difficulty in distinguishing the small bowel from the sigmoid in cases with complex anatomical relationships. In such cases, D_Pred_ for the small bowel may have been overestimated due to the misclassification of the sigmoid colon as small bowel. Nevertheless, since no statistically significant differences were observed between D_GT_ and D_Pred_ for all OARs except the small bowel, it is suggested that the impact of the inferred contours on dose evaluation was limited.

Regarding cumulative dose evaluation (Figure 5), cases in which dose constraints were exceeded for both EQD2_GT_ and EQD2_Pred_ were observed for the rectum. This may reflect actual clinical scenarios, in which treatment plans are sometimes intentionally optimized beyond predefined constraints to prioritize tumor control. Additionally, for the rectum and Rec+Sig, some patients exhibited EQD2_Pred_ values exceeding dose constraints over the three‐fraction cumulative total (Figure 5). These observations were specific to cases where the rectum was in close proximity to the HR‐CTV, making the boundary difficult to distinguish on CT images. Minor contour variations near the radioactive source may have influenced D_Pred_, and these effects likely became more pronounced when evaluated as a cumulative dose across all three fractions.

Comparatively, Mohammadi et al.^16^ reported ΔD_2cc_ values for bladder, rectum, and sigmoid as −0.50 Gy, 0.32 Gy, and −0.47 Gy, respectively, which are comparable to the values obtained in the present study (bladder: 0.17 Gy, rectum: 0.014 Gy, sigmoid: −0.073 Gy). On the other hand, Lei et al.^19^ reported ΔD_2cc_ values for bladder, small bowel, rectum, and sigmoid as 0.26 Gy, 0.11 Gy, 0.21 Gy, and 0.08 Gy, respectively. Although our study demonstrated comparable contouring accuracy (Table 2), the ΔD_2cc_ values tended to show larger discrepancies. Geometric agreement metrics, such as DSC and HD, evaluate the overall structural consistency and do not adequately reflect contour discrepancies of a few voxels in high‐dose regions near the source. Therefore, even when the global agreement is high, these local contour deviations may result in dose differences.

We also evaluated the impact of the presence or absence of combined interstitial needles on automated contouring accuracy using geometric metrics (Table 3) and the dosimetric metric ΔD_2cc_ (Table 4). First, in the geometric evaluation (Table 3), no statistically significant differences were observed between the needle and non‐needle groups for all OARs (*p* > 0.05). This indicates that the model can maintain a stable contouring accuracy remarkably unaffected by the presence of needles. On the other hand, for Rec+Sig, the values of DSC, sDSC, and 95HD tended to improve in the group with needles. Similarly, higher values were observed for the small bowel and sigmoid in the group with needles compared to those without. A possible factor is that the interstitial needles, displayed as high‐intensity signals on CT scan images, functioned as effective landmarks during model inference. In particular, the insertion of needles into the tumor (non‐OAR tissue) could have facilitated the identification of non‐OAR regions, thereby validating relative anatomical structures and improving inference accuracy. This effect is believed to be particularly significant in the rectosigmoid region adjacent to the tumor. Conversely, when evaluating the rectum and sigmoid individually, the uncertainty in identifying their unclear junction was substantial, which might have made the effect statistically difficult to detect. For the bladder, the effect was likely limited due to its inherently high visibility.

Subsequently, in the dosimetric evaluation per fraction (Table 4), no statistically significant differences in ΔD_2cc_ were observed based on the presence of needles for all OARs (*p* > 0.05). Even for Rec+Sig, which showed a trend toward improved trend in geometric metrics (DSC, sDSC, and 95HD), no significant difference was observed in ΔD_2cc_. Furthermore, in cumulative dose evaluation, no statistically significant differences in ΔEQD2 were observed between cases with and without interstitial needle insertion for any of the OARs (*p* > 0.05). This might be because the improvement in contour accuracy was related mainly to the overall structure, and the impact on the localized high‐dose region (near the source) where D_2cc_ is determined was limited. These results suggest that the model can maintain generally stable contouring accuracy in clinically critical regions near the source, regardless of the presence of needles.

However, this study has several limitations that must be acknowledged. First, the number of cases was small (testing data: 20 patients, 60 cases), and the dataset was obtained from a single institution. In particular, the number of cases for certain applicator types was limited and unevenly distributed. Consequently, the generalizability of the results and the statistical robustness of inter‐applicator comparisons are limited, and future verification using larger‐scale data is required. Second, to decrease variability among observers in the training dataset (GT), a stricter and more unified contouring protocol is required. This is because variations might have occurred among observers regarding the classification of the rectosigmoid junction and whether to treat the ventral descending colon as sigmoid or small bowel when defining GT. Third, this study used existing dose distributions for dosimetric evaluation and did not perform optimization based on the predicted contours. In clinical workflows, source dwell times are optimized based on the created contours. Therefore, the D_2cc_ calculated in this study may differ from the D_2cc_ in actual treatment. Future studies must conduct dosimetric evaluations including the dose‐optimization process and integrate these with visual assessments by radiation oncologists to enable a more robust clinical verification.

## 5 CONCLUSION

This study evaluated the accuracy of automated contouring using the nnU‐Net model for OARs in 3D‐IGBT cases, including those utilizing combined interstitial needles (Hybrid BT). The automated contouring accuracy of the model was generally comparable to that reported in previous studies, while achieving rapid contouring. Further, no significant degradation in accuracy due to the use of interstitial needles was observed. In fact, the results were comparable to those obtained for non‐needle cases. Further clinical validation is required; however, these findings suggest that the proposed model is applicable to cases involving interstitial needles and can contribute to streamlining the treatment planning workflow.

## AUTHOR CONTRIBUTION

**Kirika Takahashi**: Investigation, writing—original draft, visualization. **Ken Takeda**: Conceptualization, visualization, writing—review & editing. **Hisamichi Takagi**: Methodology, software, writing—review & editing. **Akari Niiyama**: Validation. **Noriyuki Kadoya**: Resources, data curation. **Yoshiyuki Katsuta**: Resources. **Kazuhiro Arai**: Resources. **Shohei Tanaka**: Resources. **Noriyoshi Takahashi**: Resources. **Takaya Yamamoto**: Resources. **Rei Umezawa**: Resources, writing—review & editing. **Keiichi Jingu**: Project administration.

## CONFLICT OF INTEREST STATEMENT

K.J. received personal fees from Elekta K.K.
